# Regression Analysis to Identify Factors Associated with Household Salt Iodine Content at the Sub-National Level in Bangladesh, India, Ghana and Senegal

**DOI:** 10.3390/nu10040508

**Published:** 2018-04-19

**Authors:** Jacky Knowles, Roland Kupka, Sam Dumble, Greg S. Garrett, Chandrakant S. Pandav, Kapil Yadav, Baitun Nahar, Ndeye Khady Touré, Esi Foriwa Amoaful, Jonathan Gorstein

**Affiliations:** 1 Iodine Global Network, Ottawa, ON K1N 5C8, Canada; jacky@jackyknowlesconsultancy.com; 2 UNICEF, New York, NY 10017, USA; rkupka@unicef.org; 3 Statistics for Sustainable Development, Reading RG1 4QS, UK; s.dumble@stats4sd.org; 4 Global Alliance for Improved Nutrition, 1202 Geneva, Switzerland; ggarrett@gainhealth.org; 5 All India Institute of Medical Sciences, New Delhi 110029, India; cpandav@gmail.com (C.S.P.); dr.kapilyadav@gmail.com (K.Y.); 6 International Centre for Diarrhoeal Disease Research, Dhaka 1212, Bangladesh; baitun@icddrb.org; 7 Cellule de Lutte Contre la Malnutrition, B.P. 45001 Dakar-Fann, Senegal; ndeyekhadytoure@yahoo.fr; 8 Nutrition Department, Ghana Health Service, Accra, Ghana; esiforiwa@gmail.com; 9 Iodine Global Network, Seattle, WA 98107, USA

**Keywords:** iodine deficiency, iodised salt, single variable regression, multiple variable regression

## Abstract

Regression analyses of data from stratified, cluster sample, household iodine surveys in Bangladesh, India, Ghana and Senegal were conducted to identify factors associated with household access to adequately iodised salt. For all countries, in single variable analyses, household salt iodine was significantly different (*p* < 0.05) between strata (geographic areas with representative data, defined by survey design), and significantly higher (*p* < 0.05) among households: with better living standard scores, where the respondent knew about iodised salt and/or looked for iodised salt at purchase, using salt bought in a sealed package, or using refined grain salt. Other country-level associations were also found. Multiple variable analyses showed a significant association between salt iodine and strata (*p* < 0.001) in India, Ghana and Senegal and that salt grain type was significantly associated with estimated iodine content in all countries (*p* < 0.001). Salt iodine relative to the reference (coarse salt) ranged from 1.3 (95% CI 1.2, 1.5) times higher for fine salt in Senegal to 3.6 (95% CI 2.6, 4.9) times higher for washed and 6.5 (95% CI 4.9, 8.8) times higher for refined salt in India. Sub-national data are required to monitor equity of access to adequately iodised salt. Improving household access to refined iodised salt in sealed packaging, would improve iodine intake from household salt in all four countries in this analysis, particularly in areas where there is significant small-scale salt production.

## 1. Introduction

Universal salt iodisation (USI) is globally accepted as the most cost-effective public health strategy to prevent iodine deficiency. In 1994, the Joint UNICEF/WHO Committee on Health Policy recommended that all food-grade salt used by households, by food processing industries, and for animal feed; should be fortified with iodine as a safe and sustainable strategy for the prevention and control of iodine deficiency disorders (IDD) [1]. Since then, collaboration between national governments, the salt industry, international and national organizations and academia has resulted in USI being one of the greatest public nutrition successes of the past two decades. Globally, 86% of households use salt with some iodine [2]. Household use of adequately iodised salt, defined here as salt with at least 15 mg/kg iodine [3], is not included in the UNICEF report due to the fact that most data are sourced from surveys that used field-based qualitative salt iodine testing.

Despite the global success there is an increasingly apparent inequity in household access to adequately iodised salt within some countries [4,5,6]. Factors determining access have been proposed to relate to weaknesses in salt industry capacity in relation to the level of salt refinement, quality assurance and control, below optimal implementation of regulations in support of salt iodisation legislation, and low consumer awareness about the importance of using adequately iodised salt. However, a strong evidence base for which determinants are most associated with household salt iodine content, and in which context, is lacking. This gap in sub-national data presents a challenge to the design of strategies to improve equity of access to adequately iodised household salt.

Therefore, we assessed the key determinants of household salt iodine content using data from iodine surveys conducted in Bangladesh, India, Ghana and Senegal during the period December 2014 to April 2015. All four countries have mandated the iodisation of household salt, introduced during the period 1989 (Bangladesh) to 2001 (Ghana) [7,8,9,10]. In the South Asian countries of Bangladesh and India, medium to large scale salt producers account for approximately 70–80% of the estimated national salt market [11], while in the West African countries of Ghana and Senegal, this is only 30–40% of the market, the rest being supplied by small-scale salt production (Estimates developed nationally in preparation for GAIN-UNICEF USI Partnership regional workshops in Ethiopia and Manila.) Large scale producers are more likely to have the capacity to produce refined salt, which is associated with greater homogeneity of iodisation [12]. At the time of planning these surveys, the most recent data on national iodine nutrition among school-age children were: for Bangladesh, a median urinary iodine concentration (MUIC) of 146 µg/L in 2011 [13]; for India, no national iodine status data were available; for Ghana, the MUIC varied from 255 µg/L in the South zone to 166 µg/L in the middle zone and 79 µg/L in the North zone (unpublished report from a 2010 survey, Ghana Health Services); for Senegal, a national MUIC of 104 µg/L in 2010 [14].

The main objective of this paper is to present the results of regression models designed to highlight which of the survey-related variables were most associated with household access to adequately iodised salt and whether these varied within each country. 

## 2. Materials and Methods

### 2.1. Survey Design

In brief, the surveys were, cross-sectional, stratified, multistage cluster sample design with probability proportional to size (PPS) selection of the primary sampling units (PSUs) within each stratum. Strata were determined to provide representative information for administratively or programmatically relevant domains. Households were systematically, randomly, selected within a PSU. Questions were included on a variety of indicators believed to be associated with household salt iodine content and access to adequately iodised salt. The target unit was the household. The first choice of respondent was a woman responsible for organizing food preparation in the household, usually the wife of the head of household, or the head of the household. The second choice respondent was a woman of reproductive age (WRA). Although a WRA was defined as being 15–49 years of age, women aged 18 or above were preferentially selected in Ghana, India and Senegal. Another adult member of the household was selected where nobody meeting these criteria was present.

Stratification in Bangladesh, Ghana and Senegal was based on existing knowledge of the salt supply and designed to obtain representative data for areas expected to have low household coverage of adequately iodised salt. These were: in Bangladesh, low-performing areas, as defined by the national Control of IDD project, which tended to be harder-to-reach areas, border areas, and areas of small-scale seasonal salt production; in Ghana and Senegal, small-scale salt production areas, which were associated with operational challenges and leakage of unprocessed, non-iodised, salt into the local markets. In India, stratification was by urban and rural area within six defined geographical zones: South, West, Central, North, East and North-East (12 strata in total). Table 1 describes the number and type of strata included in each survey.

### 2.2. Survey Administration and Field Procedures

For all surveys, interviews were conducted in all selected and consenting households. Data collection was supervised and quality-assured by field supervisors, with coordination and technical assistance from survey management personnel at a central administrative level. All survey-related personnel were trained prior to the surveys, and survey tools and procedures were pilot-tested in a typical field setting. Apart from Bangladesh, replacement of households where a respondent was not at home or the potential respondent refused was not implemented.

According to the different national protocols, a sample of 20–50 gm of salt was collected from all consenting households in each survey. Salt samples for each household were kept in re-sealable bags coded with the unique household identification number. Bags for all household samples from each cluster were stored in opaque bags/envelopes at room temperature until analysis of the salt iodine content at a central laboratory.

In three countries (India, Ghana and Senegal), data were collected using mobile devices with pre-coded skips and cross-checks to ensure data quality. In Bangladesh, data were collected using paper forms. Data quality was ensured in all cases by random repeat interviews; by end-of-day checks and follow-up by field supervisors. Electronic data were regularly reviewed and monitored for completeness. In Bangladesh, validated double data entry with checks for valid ranges, legal values, and consistency was conducted then the two data sets were reconciled.

### 2.3. Indicators/Survey Tools

The survey questionnaires in all countries included modules to classify residence type (urban vs. rural), consent, and recording of collection of a household salt sample. Questions were also included to determine the household Multidimensional Poverty Index (MPI) score [15,16], respondent awareness of iodine deficiency and of iodised salt (having ever heard of either); the household’s typical household salt purchasing behaviour, including whether salt was obtained in a sealed pack and, if so, whether the pack had an iodine label or logo; and whether the respondent looked for iodised salt at the point of purchase.

The MPI is comprised of three scored domains, with sub-components, for each of health, education and living standards. Each domain was given an equal one-third weight in determining the overall MPI score. A household was classified as being deprived in any one domain if the score for that domain was greater than or equal to 0.3 (scale of 0 to 1). Where the overall MPI score was greater than or equal to 0.3 (scale of 0 to 1), the household was considered as being vulnerable to acute poverty.

The primary outcome indicator for the survey was household coverage of adequately iodised salt, assessed as the percent households using salt with ≥15 mg/kg of iodine. Additional salt iodine related indicators are presented in respective national survey reports (Ghana Health Services, UNICEF, GAIN National Iodine Survey Report Ghana 2015, Draft February 2017) [17,18,19].

### 2.4. Determination of Salt Iodine Content

All salt iodine results are based on quantitative analysis of salt iodine content by the iodometric titration method [3]. An external quality-assurance (QA) network was established for the duration of the surveys, with a third-party laboratory (Uttar Pradesh State USI Coalition Technical Laboratory, Department of Endocrinology and Molecular Medicine–Biotechnology. Sanjay Gandhi Postgraduate Institute of Medical Sciences, Lucknow 226 014, India) providing internal and external QA salt samples to assess the methodology and performance of the four national laboratories conducting the salt iodine analysis.

Salt grain type was assigned by the laboratory staff to reduce subjectivity that can occur with assessment by many different field staff. Grain type categories were assigned based on physical characteristics and national terminology as fine or coarse for Bangladesh, Ghana and Senegal; and as refined, washed, crystal or *phoda* (very large crystals, typically over 5 mm across) for India.

### 2.5. Data Analysis

The Statistical Services Centre (SSC), University of Reading, United Kingdom conducted the initial survey data management and analyses for surveys in Ghana and Senegal; and provided technical support to the International Centre for Diarrhoeal Disease Research (icddr.b) and the All India Institute of Medical Sciences (AIIMS), New Delhi for these processes for surveys in Bangladesh and India respectively. All remaining analyses for this manuscript were conducted by one of the paper authors (SD). Regression models were built with inclusion of the three domain-specific MPI scores for education, health and living standards, instead of the overall MPI score, to allow for more refined analysis of factors associated with salt iodine content. All analyses were conducted separately for each country.

Results are presented for the percent households using adequately iodised salt (≥15 mg/kg iodine) at the time of the interview, the median and mean salt iodine content from all households with salt samples along with the inter-quartile range (IQR) and the 95% confidence interval (CI) around the median and mean, respectively. All data presented in this paper are for households with a valid salt iodine result, weighted for the relative proportion of the population in each stratum.

Salt iodine content was analysed against multiple different factors using general linear models, with household weights and robust variance estimation accounting for survey design effects using the survey library “Survey: analysis of complex survey samples” within the R statistical analysis package version 3.31 [20]. *p*-Values presented are based on the Wald test determining the overall significance of each variable. Significance was set as a *p*-value <0.05.

For single variable regression analysis, *p*-values are not adjusted for multiple comparisons; many of the factors considered are explicitly non-independent (e.g., Strata and urban/rural) so a naïve adjustment measure would not be appropriate. However, the number of tests being conducted should be taken into consideration when considering the significance of variables with borderline *p*-values, between 0.01 and 0.05.

For multiple variable regression analysis, all variables included in the single variable analysis were considered for inclusion in the models except for residence type where this was included in the definition of the strata (as was the case in Bangladesh, India, and Senegal). A stepwise selection procedure was conducted using a *p*-value of 0.1 as the inclusion criteria. Differences were represented by the *p*-value for variable effect. The final model included interaction by strata to investigate if and how associations between variables and salt iodine content varied by strata. In India, this was modelled using interaction by urban/rural residence and interaction by zone to simplify interpretation and graphical representation. The significance of differences were represented by the *p*-value for interaction with strata/zone effect and also graphically as the estimated household salt iodine content (geometric mean with 95% confidence intervals around the estimate) for some of the significant (*p* < 0.05) associations for each country.

The national surveys were approved by national or academic Institutional Review Boards in each of the four countries. All protocols required consent for the interview and for collection of a salt sample. Further details of individual survey design, tools, and data management, adjustments, and analysis can be found in the full survey reports [18,19,21] (personal communication from Ghana Health Services on the unpublished draft survey report).

## 3. Results

An overview of the survey design, response rates for completed interviews and salt iodine analysis, and the type of respondent (sex and age group) for each of the four countries are included in Table 1. Response rates were over 90% for completed interviews in all countries and for salt iodine analysis in India and Bangladesh. In Ghana and Senegal, response rates for salt iodine analysis were 79.6% and 74.3% respectively.

### 3.1. Single Variable Regression Analyses and Household Iodised Salt Coverage

Results for household coverage with adequately iodised salt and results of the single and multiple variable regression analyses are shown in Table 2, Table 3, Table 4 and Table 5 for Bangladesh, India, Ghana and Senegal respectively.

Strata-specific mean household salt iodine and household coverage with adequately iodised salt was found to be lowest in areas that included significant levels of small scale salt production in Bangladesh, Ghana and Senegal; and highest in more urbanised areas of all four countries.

For each of the four countries, single variable regression analysis showed that household salt iodine content was significantly different between strata (*p* < 0.001), and was significantly higher among households: with non-deprived MPI living standards (*p* ≤ 0.002), with a respondent who had heard of iodised salt (*p* ≤ 0.013), with a respondent who looked for iodised salt at the time of purchase (*p* < 0.001), where salt was obtained in a sealed pack (*p* < 0.001), and where the salt was of a more refined grain type (*p* < 0.001). For three of the four countries, household salt iodine content was significantly higher among: households of urban residence type (*p* ≤ 0.014; Bangladesh, India and Senegal); households non-deprived in the MPI Health domain (*p* ≤ 0.035; Bangladesh, Ghana and Senegal); where salt was from a major brand, and where the salt packaging had an iodine label or logo (*p* < 0.001; India, Ghana and Senegal for both, however, questions about salt brand and logo were not included in the Bangladesh survey tool). In Ghana and Senegal, household salt iodine was also found to be significantly higher among households non-deprived in the MPI education domain (*p* ≤ 0.028).

Despite the significant differences in salt iodine content between the 12 strata from the India survey, the mean household salt iodine content was relatively consistent and adequate within all strata: the mean (and 95% CI) ranged from 19.7 mg/kg (16.3, 23.2) in the South-rural stratum to 30.4 mg/kg (28.9, 31.8) in the North-urban stratum. Respective strata level household coverage with adequately iodised salt were 55.4% (95% CI 46.3%, 64.6%) and 95.9% (95% CI 93.6, 98.3). The mean household salt iodine was above 15 mg/kg for almost all variable sub-groups in India, except where salt was not obtained in a sealed pack (mean salt iodine content 14.4 mg/kg 95% CI 12.0, 16.9), and where the salt grain type was crystal or *phoda* (mean salt iodine 9.5 mg/kg 95% CI 7.8, 11.1).

For Bangladesh and Ghana, the household salt iodine content varied greatly by strata and by variable sub-group. In Bangladesh, the mean (and 95% CI) iodine content by strata ranged from 12.4 mg/kg (9.6, 15.2) in the rural-low-performing stratum to 24.3 mg/kg (20.4, 28.2) in the urban stratum. Respective strata level household coverage with adequately iodised salt were 25.1% (95% CI 14.7%, 35.6%) and 68.7% (95% CI 57.0%, 80.4%). In Ghana, the mean (and 95% CI) iodine content by strata ranged from 13.8 mg/kg (10.9, 16.7) in the South-salt-producing stratum to 32.3 mg/kg (25.2, 39.4) in the South non-salt-producing stratum for Ghana. Respective strata level household coverage with adequately iodised salt were 19.3% (95% CI 14.1%, 24.4%) and 48.6% (95% CI 38.2%, 59.0%). Salt iodine was particularly low (mean <10.0 mg/kg) among households where salt was of a coarse grain type (mean 9.4 mg/kg; 95% CI 7.0, 11.8, and mean 6.1 mg/kg; 95% CI 5.3, 6.9) in Bangladesh and Ghana respectively, and, in Bangladesh, where salt was not obtained in a sealed pack (mean 4.7 mg/kg 95% CI 4.2, 5.3). In both countries, the highest mean household salt iodine content was found for households where the respondent had looked for iodised salt at purchase: mean 32.0 mg/kg (95% CI 29.2, 34.7) in Bangladesh and mean 45.6 mg/kg (95% CI 40.6, 50.6) in Ghana.

In Senegal, household salt iodine content was consistently low by strata and variable sub-group. The mean (and 95% CI) by strata ranged from 8.8 mg/kg (6.9, 10.7) in the rural-salt-producing stratum to 17.8 mg/kg (16.0, 19.6) in the urban stratum. Respective strata level household coverage with adequately iodised salt were 10.9% (95% CI 6.7%, 17.1%) and 53.3% (95% CI 46.1%, 60.5%). There were no sub-groups in Senegal where the mean household salt iodine was ≥20 mg/kg. The highest mean salt iodine was found among households using fine grain salt (mean 19.5 mg/kg; 95% CI 16.3, 22.7).

### 3.2. Multiple Variable Regression Analyses

Multiple variable regression analyses showed that the iodine content was significantly higher in a more refined salt grain type in all four countries (*p* value variable effect <0.001). Relative to the reference (coarse grain salt), salt iodine content ranged from 1.3 (95% CI 1.2, 15.5) times higher for fine salt in Senegal to 6.5 (95% CI 4.9, 8.8) times higher for refined salt in India. See Table 2, Table 3, Table 4 and Table 5.

In India, Ghana and Senegal, the association between household salt iodine content and strata was still significant in the multiple variable regression (*p* value variable effect <0.001). In India, Central-rural was the only stratum where household salt iodine was significantly higher (1.5 times 95% CI 1.2, 1.8) than in the reference stratum of South-urban. Other variables that showed a significant association with higher household salt iodine were: the respondent looking for iodised salt at the time of purchase, 1.6 times (95% CI 1.2, 2.0) in Bangladesh and 1.7 times (95% CI 1.4, 2.0) in Ghana; obtaining salt in a sealed pack, 3.8 times (95% CI 2.9, 5.0) higher than loose salt in Bangladesh; using salt from a leading market brand, 1.5 times (95% CI 1.2, 1.9) higher than for no brand in India; and households being non-deprived in the MPI health domain, 1.1 times (95% CI 1.0, 1.2) higher than salt in households deprived in the MPI health domain in India.

Introducing the additional interaction with strata (separately for residence type and zone rather than by strata for India) into the model revealed new results. The significance of these interactions are shown in the last column of Table 2, Table 3, Table 4 and Table 5. Including interactions by urban/rural residence in the model for India showed non-significant differences for all variables, therefore, Table 3 and the results below only include the interaction by zone.

**Figure 1:**
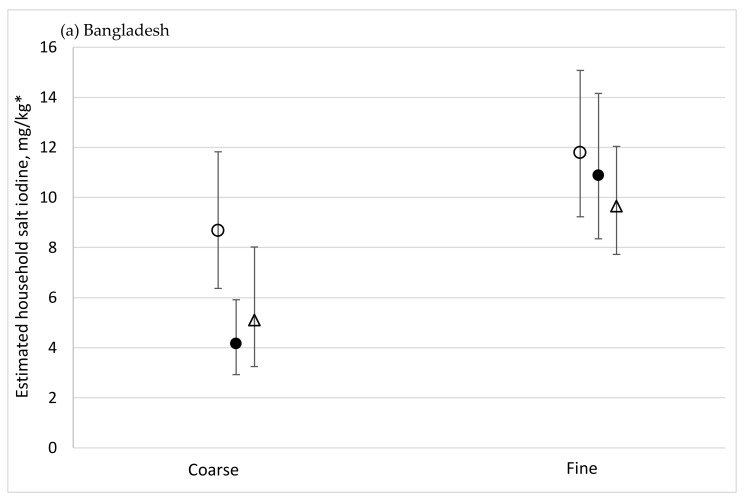

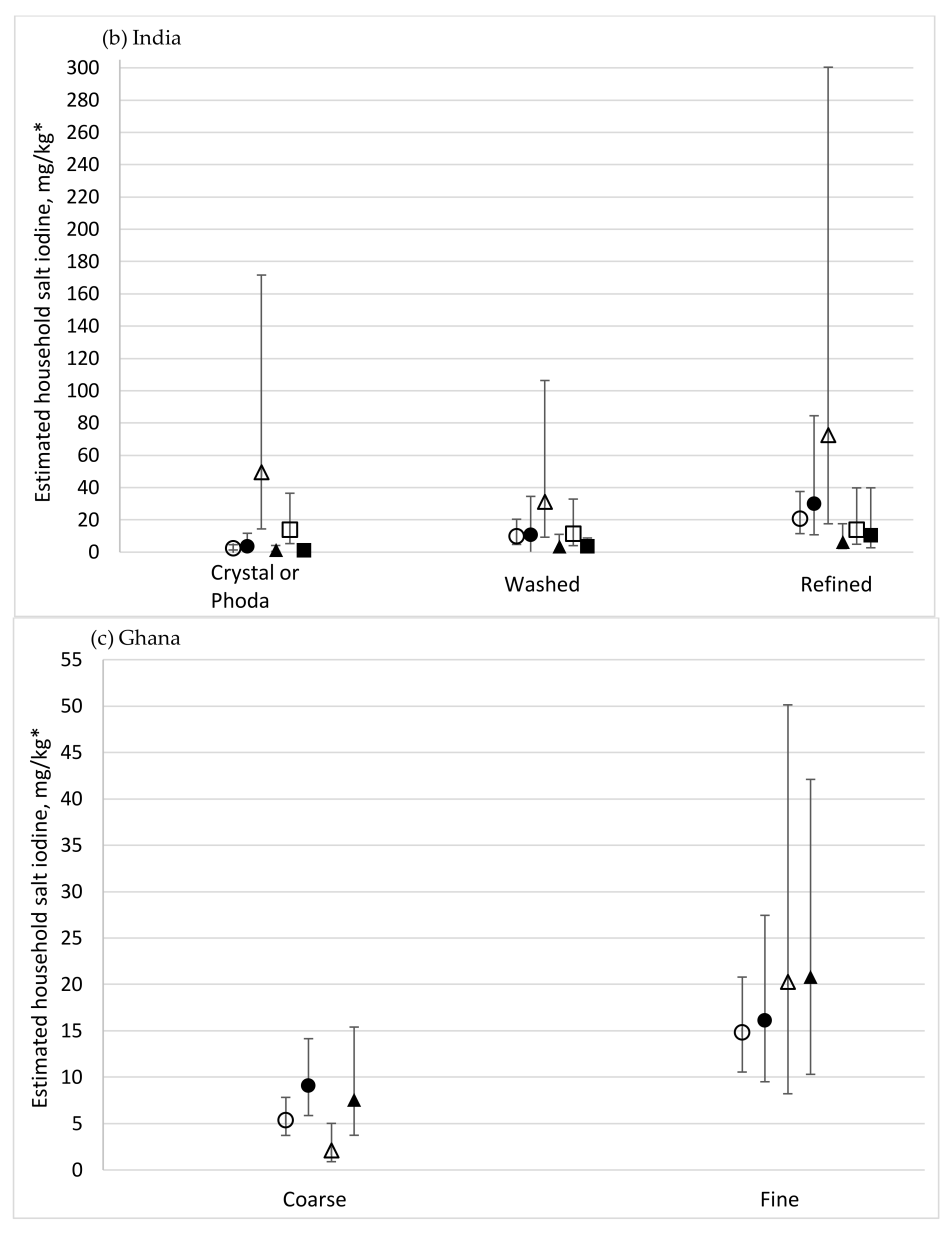

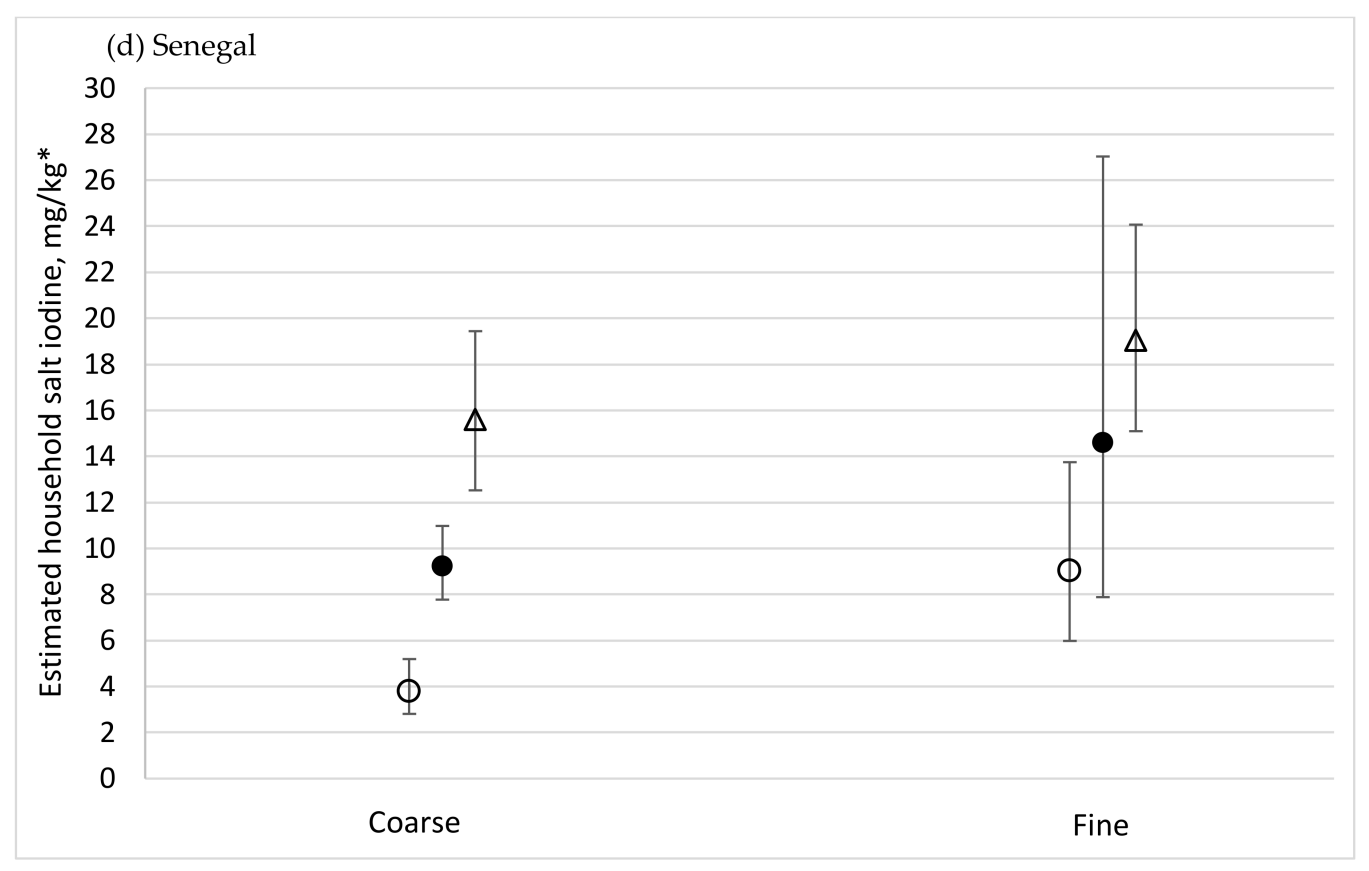
Multiple regression analysis including interaction with strata, to estimate household salt iodine (mg/kg) according to salt grain type in (**a**) Bangladesh, ○ Rural low-performing, ● Rural other, ∆ Urban; (**b**) India, ○ South, ● West, ∆ Central, ▲ North, □ East, ∎ North East; (**c**) Ghana, ○ South-salt-producing, ● North, ∆ Mid, ▲ South-non-salt-producing; and (**d**) Senegal. ○ Rural-salt-producing, ● Rural-non-salt-producing, ∆ Urban. * Adjusted estimates with 95% confidence intervals for salt iodine content, back-transformed from a log-linear model adjusting for: urban/rural residence, multi-dimensional poverty index (MPI) components, awareness of iodised salt, awareness of iodine deficiency, salt brand, iodine label or logo on packaging (except Bangladesh), and respondent looked for iodised salt at purchase.

**Figure 2:**
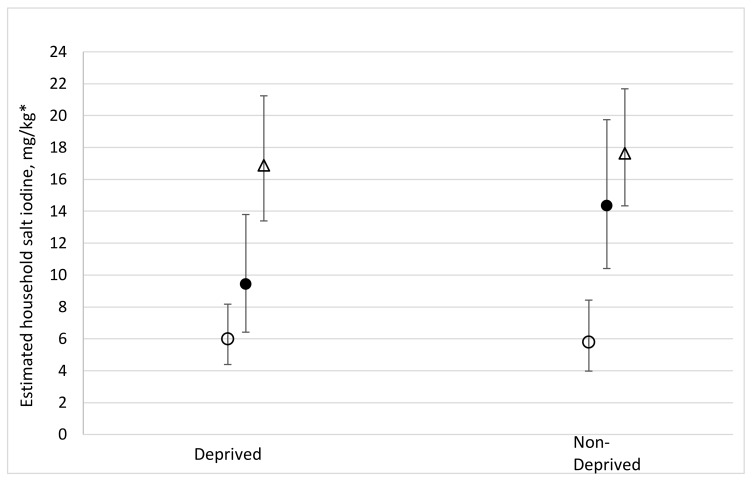
Multiple variable regression analysis including interaction with strata in Senegal; ○ Rural-salt-producing, ● Rural-non-salt-producing, ∆ Urban; to estimate household salt iodine (mg/kg) according to household multi-dimensional poverty index (MPI) (living standards). * Adjusted estimates with 95% confidence intervals for salt iodine content, back-transformed from a log-linear model adjusting for: urban/rural residence, MPI components (education and health/nutrition), awareness of iodised salt, awareness of iodine deficiency, salt brand, iodine label or logo on packaging, respondent looked for iodised salt at purchase, and grain type.

Statistically significant interactions that could be meaningfully interpreted are presented in Figure 1 and Figure 2.

Significant differences in the strata/zone level interaction between salt grain type and estimated household salt iodine were found in Bangladesh (*p* = 0.030), India (*p* < 0.001), Ghana (*p* < 0.001), and Senegal (*p* = 0.011). For Bangladesh, the only pairwise significant difference in estimated household salt iodine content (based on non-overlapping 95% confidence intervals shown in Figure 1a) was between households using coarse grain salt in the rural-low-performing stratum (higher iodine after holding other factors constant) and households using coarse grain salt in the rural-other stratum. Salt from households using fine grain salt had similar estimated iodine levels in all three strata. In India, estimated household salt iodine in the Central zone was higher than the estimated level in other zones for all grain types, however, only significantly higher among households using crystal/phoda salt when compared with all other zones except for the East (Figure 1b). The strata level interaction between grain type and estimated household salt iodine in Ghana was only significantly different between households using coarse salt in the North (significantly higher estimated salt iodine) and in the Mid strata (Figure 1c). In Senegal, Figure 1d indicates that for households using salt with a coarse grain type, the estimated household salt iodine content was significantly different between all strata, with highest levels in the urban stratum and lowest levels in the rural-salt-producing stratum. The estimated salt iodine content for fine grain salt was also significantly higher among households in the urban stratum compared with households in the rural-salt-producing stratum. The iodine level of fine grain salt used by households in the rural-non-salt-producing stratum showed wide variation and was not significantly different to the levels found for the other two strata.

Other than for some of the packaging-related variables, the only other significant differences found for strata level interactions with estimated household salt iodine were for the MPI health domain in Bangladesh (*p* = 0.013) and for the MPI living standards domain in Senegal (*p* = 0.036). For Bangladesh the sample size for households deprived in this domain was small, *n* = 132 nationally, making the one pairwise difference between estimated salt iodine from rural-low-performing (higher iodine) and rural-other households difficult to interpret. Figure 2 shows that, in Senegal, the estimated household salt iodine among deprived and non-deprived households (for the MPI living standards domain) was approximately the same when compared within each of the urban and rural-salt-producing strata, although the level was significantly higher among households in the urban stratum. Within the rural-non-salt-producing stratum, the estimated salt iodine content was not significantly higher among households that were not deprived in the MPI living standards domain, but the estimated salt iodine content among not deprived households was significantly higher than in the rural-salt-producing stratum.

## 4. Discussion

The national surveys in all four countries were designed to investigate the coverage of adequately iodised household salt as well as the levels of iodine in the salt. This paper assessed the relationship between household salt iodine content and a number of factors previously found to be associated with household access to adequately iodised salt. In single variable regression analyses, salt iodine content was significantly associated with indicators of: location (strata), socio-economic status (particularly the MPI living standards domain), knowledge and awareness of iodised salt (having heard of it and looking for it at the point of purchase), and salt supply (salt obtained in a sealed pack and salt grain type). The results of the multiple variable regression analysis provide a clear indication that various characteristics of the salt supply are the most important factors associated with household salt iodine level. In particular, the level of iodine in refined salt was considerably higher than in larger coarse and crystal salt. These results also highlight the high level of sub-national variation in the quality of salt accessible to households.

The tendency for lower income, rural, households to have greater access to non-iodised or inadequately iodised, presumably lower-priced, salt has been reported previously [4,6,22,23]. A notable outcome of the multiple variable regression analyses was that in all countries, the significance of any positive association between respondent awareness of iodine deficiency and iodised salt with salt iodine content was removed after the influence of supply-related factors were accounted for. This does not mean that supply-chain and consumer-focused communication activities are not an important part of a programme to achieve optimal iodine nutrition through USI. However, it indicates that demand-side activities such as communication and awareness raising, in the absence of concurrent interventions to strengthen supply-side awareness and practices, are unlikely to be the main factor driving household access to adequately iodised salt. This finding has been reported elsewhere [24,25].

Another main finding was that the highly significant single variable associations between salt iodine and salt supply-related factors, such as packaging and brand name, were generally reduced when grain type was held constant, suggesting an overlap between these factors, as would be expected. Experience has shown that salt with a finer grain type is more likely to be: iodised homogenously, subject to production quality control and regulatory monitoring, packaged and labelled as iodised, and to be produced by a larger scale producer with a known brand name [12].

The low household access to adequately iodised salt in small-scale salt producing areas of Bangladesh, Ghana and Senegal indicates a lack of progress following efforts, over many years, to improve salt iodisation technology and quality assurance capacity among small-scale producers [12,26,27,28]. These areas had higher household access to coarse salt (66.2%, 69.0% and 78.6% respectively) than in other strata within the same country (data not shown). Across all four surveys, household salt with lower iodine content was generally found to be unrefined with a larger crystal size, which is typically produced by smaller-scale enterprises. Smaller production units often face technical and financial challenges to integrate quality-assured iodisation into the production process, although this has been done relatively successfully in some contexts [29]. For a number of reasons, including costs and logistics, local authority officials are also less likely to implement national guidelines for regulatory monitoring within the decentralised, often seasonal, environment of small-scale salt production (Yusufali R, Situma R, Bohac L, Khan L and J Gorstein. A Review of Country Experiences in Small-Scale Salt Iodization. Submitted for publication) [12].

As previously reported [6], India has made impressive progress towards achieving USI. In fact, the goal of ≥90% household coverage has already been achieved in the urban areas of the Central, North and North-East zones. This was reflected in the urinary iodine results from the 2015 survey that showed at the national level WRA had adequate iodine status (MUIC 158 µg/L) [30]. The WHO recommended cut-off indicating adequate iodine status is a population MUIC of 100 µg/L, however some research indicates this should be lower for WRA [31]. It appears that national success in salt iodisation is in part due to the Salt Department strategy to consolidate the industry and improve quality assurance and regulatory monitoring procedures. Based on the higher iodine content noted in refined, packaged salt and the outcome of the multiple variable analysis, it could be presumed that if these same strategies are continued and expanded to remaining areas of the country, coverage should improve in the South and rural-Central areas and the target of 90% national household coverage with adequately iodised salt will be achievable. However, the recent policy decision to close down the Salt Commissioner’s Department may adversely affect the success story of USI in India.

In Bangladesh, the survey report [21] and the outcome of the multiple variable regression analysis indicated that iodisation of salt from small-scale production may be more operational than in Ghana or Senegal. A higher proportion of coarse salt is packaged in Bangladesh than in the other two countries and packaged coarse salt was more likely to be iodised than loose coarse salt. Despite this, there is still evidence of overall low household coverage with adequately iodised salt in the rural-low-performing areas and of low median and mean iodine content of coarse salt. The multiple variable regression indicated that the variable “respondent looked for iodised salt” remained significantly associated with household salt iodine, which is an encouraging indication that consumer awareness may influence choice in this country. However, only about 10% (179 respondents) of the total weighted sample reported to have looked for iodised salt at purchase. The 2015 survey in Bangladesh did not include assessment of iodine status. However, the 2011 survey results indicated a high level of association between household salt iodine and population iodine status. This suggests that the population in areas of the country where lower quality, coarse, salt is readily available may have lower, possibly inadequate, iodine intake.

In Ghana, the higher iodine content of coarse salt in the North compared with the Mid strata is difficult to explain, however, may be related to the expectation of increased regulatory monitoring check points during the longer journey to retail points in the North. Despite this difference, the fact that the mean iodine level of coarse salt was below 10 mg/kg in all four strata indicates that a similar strategic response is required to ensure that this type of salt is adequately iodised. The national MUIC among WRA of 202 µg/L indicates adequate iodine intake among this population group, suggesting that sources of dietary iodine, other than iodised household salt, are available (personal communication from Ghana Health Services on the unpublished draft survey report). Sources could be naturally occurring iodine and/or iodised salt used in frequently consumed processed foods, such as bouillon and tomato paste. One study conducted in the North of Ghana supports this hypothesis [32].

In Senegal, it appears that within each of the urban and rural-salt-producing strata, access to iodised household salt was consistent. Within the urban stratum, the majority of households were using salt with just about adequate iodine content, whereas in the rural-salt-producing stratum, household salt tended to have very low estimated iodine content. In contrast, households in the rural non-salt-producing stratum were accessing salt with either relatively high or else low level of iodine. The MPI living standards score appeared to be a key driver of access to better iodised salt in this stratum, suggesting that salt of varying quality was available, but that access to higher quality iodised salt was associated with salt pricing/affordability. Until the challenge of small-scale supply of lower-iodisation quality salt is addressed, it is unlikely that much can be done to influence the effect of the socio-economic factor on household salt use. In contrast to the findings in Ghana, the low percent access to adequately iodised salt was reflected in a relatively low MUIC (98 µg/L) among WRA nationally [19]. 

The iodine content of household salt is an important indication of iodine intake from salt iodisation, however, there is now a large body of evidence indicating that industrially-processed food salt is an increasingly important source of, potentially iodised, salt [33,34]. Food industry salt is included in the definition of USI. This manuscript presents evidence of large variations in access to adequately iodised household salt and highlights some of the challenges to improving this situation. It is, therefore, recommended that USI implementing guidelines should be expanded to include enforcement of the use of adequately iodised salt by the processed food and condiment industry to improve population-wide access to salt iodine.

It was possible to apply the same regression model in each country and conduct some comparison between them because they were designed with similar questionnaire modules. In addition, the stratification design and multiple regression analyses provided results that can be used as an evidence base for future programme strategies to increase equity of access to adequately iodised salt. A national process for dissemination and strategic use of these findings has been encouraged in all four countries.

The external quality assurance of laboratory performance rated all four laboratories conducting salt iodine analyses as “Good”, providing confidence in the salt iodine results.

A limitation to the sample size in Senegal occurred due to the misplacement of salt samples from 13 PSUs across different strata. Salt iodine data were adjusted for this since the occurrence could not be considered as random non-response. The non-response for valid household salt iodine results in Ghana was random and more related to households that typically purchased small quantities of salt and not having any in the household at the time of the survey, and to collection of insufficient salt for analysis.

## 5. Conclusions

Disparities in household access to adequately iodised salt has been a common observation in a number of countries, even after a period of years implementing salt iodisation. This awareness reinforces the importance of collecting representative sub-national data and using these data to improve programme performance and ensure that all segments of populations are reached with sufficient iodine through adequately iodised salt to meet minimum physiological needs. Designing surveys with standardised modules, programme-related stratification, quantitative assessment of salt iodine, and using multiple variable regression as part of the data analysis, can greatly improve this sub-national evidence base. It also provides a baseline from which to monitor the impact of implementation.

Improving household access to quality-assured refined iodised salt in sealed packaging, would improve dietary iodine intake from household salt in all sub-national areas included in this analysis, in particular in areas of small-scale salt production. Based on a review of many initiatives to improve the quality of small-scale iodised salt production [12], it is likely that this would require at the very least a consolidation of small-scale salt processors into financially viable operations with increased capacity for internal quality control and strengthened government regulatory monitoring of the product. Until these actions are taken up and sustained, targeted iodine interventions may be required for populations known to access non-iodised or lower quality unrefined household salt produced by small-scale salt producers and which have low intakes of other sources of iodine in the diet.

## Figures and Tables

**Table 1:** Overview of the survey design, response rate and respondent characteristics for each country.

Country	Year	Target Sample Size—HHs (HHs/PSU)	Sample Design		Response Rate		Respondent Characteristics	
			Stratification	Sampling Scheme	Completed Interviews	Salt Iodine Result	Female	WRA (% 15–17 yo)
Bangladesh	2015	1512 (12)	3 strata: Urban (including slum), Rural other (non-low performing), Rural low-performing	Cross-sectional cluster, based on Multiple Indicator Cluster Survey 2009 sampling frame *With replacements* (*99 HHs*)	100.0	99.0	99.8	91.9 (2.4)
India	2014–2015	6048 (12)	12 strata: Urban/Rural by 6 zones: North, North-East, East, West, Central, South	Cross-sectional cluster, PPS within strata *Without replacements*	94.5	93.9	91.3	82.2 (0.7)
Ghana	2015	2112 (16)	4 strata: North, Mid, South non-salt-producing, South salt-producing	Cross-sectional cluster, PPS within strata *Without replacements*	91.3	74.3	83.8	61.4 (0.0)
Senegal	2014	1968 (16)	3 strata: Urban, Rural non-salt-producing, Rural salt-producing	Cross-sectional cluster, PPS within strata *Without replacements*	98.8	79.6	99.0	90.2 (1.4)

HH, household. PPS, probability proportional to size. PSU, primary sampling unit. WRA, woman of reproductive age. yo, years old.

**Table 2:** Regression analyses of factors associated with household salt iodine content—Bangladesh.

					Single Variable Model—Salt Iodine mg/kg					Multiple Variable Model—Salt Iodine Level			
Variable	Level	Number of HHs	% HHs with Salt Iodine > 15 mg/kg	95% CI	Median	IQR	Mean	95% CI	*p* Value	Relative to Reference	95% CI	*p* Value (Variable Effect)	*p* Value (Interaction with Strata)
Strata	Rural low-performing	495	25.1	14.7, 35.6	5.1	3.0, 15.4	12.4	9.6, 15.2	<0.001			0.111	
	Rural other	502	53.6	42.1, 65.0	20.2	3.4, 34.0	19.9 ^a^	16.4, 23.4		0.8	0.6, 1.0		
	Urban	501	68.7	57.0, 80.4	26.7	4.2, 37.0	24.3 ^a^	20.4, 28.2		1.0	0.7, 1.3		
Residence Type	Urban	501	68.7	57.0, 80.4	26.7	4.2, 37.0	24.3	20.4, 28.2	0.014	*Not included in multiple variable analysis*			
	Rural	997	44.3	34.3, 54.3	9.4	3.3, 32.6	17.5	14.6, 20.4					
MPI Education	Deprived	1007	47.4	37.8, 57.0	11.1	3.3, 33.9	18.5	15.6, 21.3	0.256			0.780	0.357
	Not deprived	491	57.5	49.0, 66.0	22.0	3.8, 33.9	20.9	18.2, 23.7		1.0	0.8, 1.2		
MPI Health	Deprived	132	39.0	26.5, 51.5	5.8	3.0, 27.0	14.5	11.2, 17.9	0.035			0.951	0.013
	Not deprived	1335	51.5	42.9, 60.0	17.8	3.4, 33.9	19.7	17.1, 22.2		1.0	0.8, 1.3		
MPI Living Standards	Deprived	1228	45.6	36.8, 54.5	10.2	3.3, 32.6	17.9	15.4, 20.5	0.002			0.669	0.629
	Not deprived	270	72.1	62.3, 81.8	28.8	4.8, 36.9	25.0	22.1, 27.9		1.0	0.8, 1.2		
Heard of iodine deficiency	No	394	36.5	23.4, 49.5	5.0	2.9, 26.7	14.3	10.5, 18.1	0.002			0.983	0.089
	Yes	1104	55.8	48.0, 63.7	22.4	4.0, 34.7	21.1	18.7, 23.5		1.0	0.8, 1.2		
Heard of iodised salt	No	283	31.1	17.2, 45.1	4.2	2.5, 24.1	12.7	8.6, 16.7	<0.001			0.974	0.289
	Yes	1215	56.1	48.6, 63.6	22.8	3.8, 34.7	21.1	18.8, 23.4		1.0	0.8, 1.2		
Salt obtained in sealed pack	No	403	3.5	1.0, 6.0	3.1	2.5, 4.9	4.7	4.2, 5.3	<0.001			<0.001	<0.001
	Yes	1093	71.8	63.1, 80.5	28.8	10.2, 36.8	25.8	23.1, 28.5		3.8	2.9, 5.0		
Respondent looked for iodised salt purchase	No	418	39.6	30.5, 48.7	5.9	3.0, 29.6	15.9	13.0, 18.9	<0.001			0.003	0.086
	Yes	179	87.0	81.0, 93.1	33.9	26.2, 40.3	32.0 ^a^	29.2, 34.7		1.6 ^a^	1.2, 2.0		
	Missing/Don’t know	901	49.1	37.4, 60.7	13.5	3.4, 32.6	18.5	15.2, 21.8		1.1	0.9, 1.3		
Grain type	Coarse	525	16.7	8.7, 24.8	3.8	2.5, 8.5	9.4	7.0, 11.8	<0.001			<0.001	0.030
	Fine	973	68.5	61.4, 75.7	27.6	6.2, 36.3	24.5	22.2, 26.7		2.0	1.5, 2.6		

CI, confidence interval. HH, household. IQR, inter-quartile range. MPI, multi-dimensional poverty index. ^a^ Superscript letter indicates a significant difference *p* < 0.05 to the reference value (first listed level), for variables with more than 2 levels.

**Table 3:** Regression analyses of factors associated with household salt iodine content—India.

					Single Variable Model—Salt Iodine mg/kg					Multiple Variable MODEL—Salt Iodine Level			
Variable	Level	Number of HHs	% HHs with Salt Iodine > 15 mg/kg	95% CI	Median	IQR	Mean	95% CI	*p* Value	Relative to Reference	95% CI	*p* Value (Variable Effect)	*p* Value (Interaction with Zone)
Strata	South—Urban	492	69.4	62.5, 76.4	26.5	9.5, 32.8	23.8	20.6, 27.0	<0.001				
	South—Rural	491	55.4	46.3, 64.6	19.0	3.2, 31.7	19.7 ^a^	16.3, 23.2		0.9	0.7, 1.1	<0.001	
	West—Urban	483	82.1	76.1, 88.0	28.6	20.1, 32.8	26.8 ^a^	24.7, 28.9		1.0	0.8, 1.2		
	West—Rural	475	71.1	64.4, 77.8	21.3	13.8, 31.7	22.9	20.6, 25.2		1.0	0.8, 1.3		
	Central—Urban	454	90.0	86.5, 93.4	29.6	23.3, 33.9	28.4 ^a^	27.0, 29.8		1.1	1.0, 1.3		
	Central—Rural	473	67.6	60.9, 74.4	22.2	12.7, 31.7	22.5	20.7, 24.3		1.5 ^a^	1.2, 1.8		
	North—Urban	422	95.9	93.6, 98.3	31.7	26.5, 34.9	30.4 ^a^	28.9, 31.8		1.1	1.0, 1.3		N/A
	North—Rural	423	78.1	70.3, 85.8	26.5	16.9, 33.9	24.8	22.5, 27.1		0.9	0.7, 1.1		
	East—Urban	484	88.7	84.9, 92.4	28.6	21.2, 32.8	27.7 ^a^	25.8, 29.7		1.0	0.9, 1.2		
	East—Rural	488	71.7	64.3, 79.2	21.2	13.8, 30.7	23.3	20.7, 25.9		0.9	0.7, 1.1		
	North East—Urban	503	93.5	90.9, 96.0	28.6	21.2, 33.9	29.2 ^a^	26.8, 31.7		1.0	0.9, 1.2		
	North East—Rural	494	76.2	67.7, 84.6	24.3	15.9, 31.7	25.4 ^a^	22.1, 28.8		1.0	0.8, 1.2		
Residence Type	Urban	2838	86.4	84.5, 88.2	28.6	21.2, 33.9	27.7	26.7, 28.6	<0.001	*Not included in multiple variable analysis*			
	Rural	2844	69.8	66.7, 73.0	22.2	12.7, 31.7	23.1	22.0, 24.2					
MPI Education	Deprived	1453	74.8	71.8, 77.9	25.4	14.8, 32.8	25.0	23.8, 26.2	0.177			0.394	0.870
	Not deprived	4229	79.2	77.2, 81.1	26.5	16.9, 32.8	25.5	24.7, 26.2		1.0	0.9, 1.0		
MPI Health	Deprived	1561	76.2	72.7, 79.8	24.3	15.9, 32.8	25.1	23.6, 26.6	0.240			0.035	0.290
	Not deprived	4121	78.8	76.8, 80.7	26.5	16.9, 32.8	25.5	24.7, 26.2		1.1 ^a^	1.0, 1.2		
MPI Living Standards	Deprived	3418	72.9	70.4, 75.4	23.3	14.8, 31.7	24.0	23.0, 25.0	<0.001			0.167	0.586
	Not deprived	2258	85.8	83.8, 87.8	29.6	21.2, 33.9	27.4	26.5, 28.2		1.1	1.0, 1.1		
Heard of iodine deficiency	No	2540	73.1	70.3, 76.0	24.3	14.4, 31.7	23.7	22.7, 24.7	<0.001			0.722	0.722
	Yes	3142	82.0	79.9, 84.0	28.6	19.0, 33.9	26.7	25.8, 27.5		1.0	0.9, 1.1		
Heard of iodised salt	No	2096	71.9	68.7, 75.2	23.3	13.8, 31.7	23.2	22.1, 24.2	<0.001			0.632	0.498
	Yes	3586	81.6	79.7, 83.5	27.5	18.0, 33.9	26.6	25.8, 27.4		1.00	0.8, 1.1		
Salt obtained in sealed pack	No	293	38.9	31.4, 46.5	8.5	3.2, 22.1	14.4	12.0, 16.9	<0.001			0.978	0.009
	Yes	5095	80.6	78.8, 82.3	26.5	18.0, 32.8	26.1	25.4, 26.8		1.00	0.8, 1.3		
Salt package had iodine logo or label ^1^	No	218	59.0	50.8, 67.2	19.0	5.3, 26.5	19.2	16.8, 21.6	<0.001			0.110	0.006
	Yes	3883	82.6	80.6, 84.5	27.5	19.0, 33.9	26.8 ^a^	26.0, 27.7		1.2	1.0, 1.5		
	Missing/Don’t know	1581	69.5	66.0, 73.1	23.3	12.1, 31.7	22.5 ^a^	21.3, 23.7		1.1	0.9, 1.4		
Salt Brand ^1^	No brand	289	50.7	43.2, 58.2	15.9	5.3, 26.5	16.6	14.5, 18.6	<0.001			<0.001	0.002
	Other brand	1147	73.1	69.4, 76.7	21.2	14.8, 29.6	22.7 ^a^	21.6, 23.9		1.2	1.0, 1.5		
	Leading market brand	3188	87.4	85.5, 89.3	29.6	21.2, 33.9	28.6 ^a^	27.7, 29.5		1.5 ^a^	1.2, 1.9		
	Missing/Don’t know	1058	61.9	57.4, 66.4	20.1	7.4, 29.6	20.3	18.9, 21.7		1.2	0.9, 1.5		
Respondent looked for iodised salt purchase	No	1069	85.3	82.6, 87.9	29.6	21.2, 34.9	28.6	27.0, 30.2	<0.001			0.210	0.028
	Yes	1950	82.6	79.9, 85.2	27.5	19.0, 33.9	26.8 ^a^	25.7, 27.8		1.0	0.9, 1.0		
	Missing/Don’t know	2663	71.7	68.7, 74.6	23.3	12.7, 31.7	22.9 ^a^	22.0, 23.9		0.9	0.8, 1.0		
Grain type	Crystal or phoda	542	24.3	18.7, 29.9	4.2	1.1, 14.8	9.5	7.8, 11.1	<0.001			<0.001	<0.001
	Refined	3777	89.6	88.0, 91.3	29.6	21.2, 34.9	29.3 ^a^	28.5, 30.1		6.5 ^a^	4.9, 8.8		
	Washed	1321	66.2	62.1, 70.2	20.1	12.7, 27.5	20.4 ^a^	19.3, 21.4		3.6 ^a^	2.6, 4.9		

CI, confidence interval. HH, household. MPI, multi-dimensional poverty index. IQR, inter-quartile range. ^a^ Superscript letter indicates a significant difference *p* < 0.05 to the reference value (first listed level), for variables with more than 2 levels. ^1^ Only asked where salt reported to be obtained in a sealed packet.

**Table 4:** Regression analyses of factors associated with household salt iodine content—Ghana.

					Single Variable Model—Salt Iodine mg/kg					Multiple Variable Model—Salt Iodine Level			
Variable	Level	Number of HHs	% HHs with Salt Iodine > 15 mg/kg	95% CI	Median	IQR	Mean	95% CI	*p* Value	Relative to Reference	95% CI	*p* Value (Variable Effect)	*p* Value (Interaction with Strata)
Strata	South-salt-producing	431	19.3	14.1, 24.4	4.0	2.7, 9.3	13.8	10.9, 16.7	<0.001				
	North	359	37.6	28.2, 47.0	9.3	4.0, 59.3	30.3 ^a^	23.6, 37.1		1.7 ^a^	1.3, 2.8	<0.001	
	Mid	407	18.6	13.0, 24.1	5.3	2.7, 10.6	15.5	11.8, 19.1		0.8 ^a^	0.6, 1.0		
	South-non-salt producing	372	48.6	38.2, 59.0	13.3	4.0, 55.9	32.3 ^a^	25.2, 39.4		1.4 ^a^	1.1, 1.8		
Residence Type	Rural	572	25.2	16.7, 33.6	6.7	2.7, 16.0	19.1	14.3, 23.8	0.400			0.300	0.413
	Urban	997	31.4	26.2, 36.7	6.7	2.7, 30.6	23.5	19.7, 27.3		0.9	0.7, 1.1		
MPI Education	Deprived	654	23.6	18.4, 28.8	5.3	2.7, 13.3	18.2	15.0, 21.4	0.028			0.740	0.154
	Not deprived	915	32.8	28.1, 37.6	6.7	2.7, 35.9	24.3	21.0, 27.6		1.0	0.9, 1.1		
MPI Health	Deprived	877	24.6	20.1, 29.2	5.3	2.7, 14.6	18.4	15.5, 21.3	0.008			0.639	0.566
	Not deprived	688	33.8	28.0, 39.6	8.0	2.7, 38.6	25.4	21.3, 29.4		1.0	0.9, 1.2		
MPI Living Standards	Deprived	1401	27.0	23.0, 31.0	6.7	2.7, 17.3	20.2	17.6, 22.8	<0.001			0.276	0.703
	Not deprived	167	46.2	36.4, 55.9	14.5	4.0, 67.6	35.5	27.5, 43.5		1.1	0.9, 1.4		
Heard of iodine deficiency	No	976	24.0	19.5, 28.5	5.3	2.7, 13.3	18.7	15.8, 21.6	<0.001			0.909	0.677
	Yes	593	36.9	31.5, 42.3	9.3	4.0, 45.9	26.7	22.8, 30.5		1.0	0.9, 1.2		
Heard of iodised salt	No	291	18.1	11.8, 24.5	5.3	2.7, 9.3	14.9	11.1, 18.7	<0.001			0.526	0.192
	Yes	1278	31.7	27.2, 36.2	8.0	2.7, 30.8	23.5	20.4, 26.6		1.0	0.8, 1.1		
Salt obtained in sealed pack	No	1009	15.1	11.2, 18.9	5.3	2.7, 9.3	13.0	10.7, 15.4	<0.001			0.799	0.011
	Yes	550	59.0	53.4, 64.7	26.6	8.0, 75.3	40.5	35.9, 45.1		1.1	0.6, 1.8		
Salt package had iodine logo or label ^1^	No	84	30.7	18.0, 43.4	5.3	2.7, 22.9	23.4	12.6, 34.2	<0.001			0.773	0.564
	Yes	396	64.7	58.6, 70.9	33.3	9.3, 77.9	43.5 ^a^	38.4, 48.5		1.1	0.6, 1.8		
	Missing/Don’t know	1089	16.5	12.5, 20.4	5.3	2.7, 9.3	14.2	11.8, 16.6		1.2	0.7, 2.2		
Salt Brand ^1^	No brand	66	14.9	4.8, 25.1	5.3	2.7, 9.3	10.9	5.6, 16.2	<0.001			0.199	0.004
	Other brand	50	59.2	42.7, 75.6	30.6	4.2, 58.6	39.7 ^a^	26.1, 53.3		1.3	0.7, 2.4		
	Leading market brand	392	65.0	58.8, 71.1	35.9	9.3, 79.8	44.6 ^a^	39.2, 49.9		1.6	1.0, 2.8		
	Missing/Don’t know	1061	16.0	12.0, 19.9	5.3	2.7, 9.3	13.7	11.3, 16.0		1.0	0.5, 1.9		
Respondent looked for iodised salt purchase	No	1068	15.1	11.5, 18.7	5.3	2.7, 9.3	12.6	10.4, 14.8	<0.001			<0.001	0.086
	Yes	426	65.5	60.0, 71.0	37.2	9.3, 79.8	45.6 ^a^	40.6, 50.6		1.7 ^a^	1.4, 2.0		
	Missing/Don’t know	75	27.9	15.4, 40.5	8.0	4.0, 26.0	22.5 ^a^	13.7, 31.4		1.1	0.8, 1.5		
Grain type	Coarse	780	5.9	3.4, 8.5	4.0	2.7, 5.7	6.1	5.3, 6.9	<0.001			<0.001	<0.001
	Fine	789	45.2	38.3, 52.1	12.0	5.3, 61.2	32.8	28.2, 37.4		2.7	2.2, 3.3		

CI, confidence interval. HH, household. MPI, multi-dimensional poverty index. IQR, inter-quartile range. ^a^ Superscript letter indicates a significant difference *p* < 0.05 to the reference value (first listed level), for variables with more than 2 levels. ^1^ Only asked where salt reported to be obtained in a sealed packet.

**Table 5:** Regression analyses of factors associated with household salt iodine content—Senegal.

					Single Variable Model—Salt Iodine mg/kg					Multiple Variable Model—Salt Iodine Level			
Variable	Level	Number of HHs	% HHs with Salt Iodine > 15 mg/kg	95% CI	Median	IQR	Mean	95% CI	*p* Value	Relative to Reference	95% CI	*p* Value (Variable Effect)	*p* Value (Interaction with Strata)
Strata	Rural-salt-producing	568	10.9	6.7, 15.1	4.9	3.1, 8.1	8.8	6.9, 10.7	<0.001				
	Rural-non-salt-producing	524	19.1	11.9, 26.3	6.9	4.6, 11.2	10.7 ^a^	8.4, 13.0		1.4 ^a^	1.2, 1.6	<0.001	
	Urban	474	53.3	46.1, 60.5	15.9	8.7, 22.8	17.8 ^a^	16.0, 19.6		2.1 ^a^	1.9, 2.4		
Residence Type	Urban	474	53.3	46.1, 60.5	15.9	8.7, 22.8	17.8	16.0, 19.6	<0.001	*Not included in multiple variable analysis*			
	Rural	1092	19.0	11.9, 26.1	6.9	4.6, 11.2	10.7	8.1, 12.9					
MPI Education	Deprived	1127	32.2	26.3, 38.1	8.8	5.4, 18.0	13.1	11.5, 14.7	<0.001			0.315	0.397
	Not deprived	439	48.1	41.2, 55.1	14.3	7.9, 22.4	17.6	15.6, 19.5		1.1	1.0, 1.2		
MPI Health	Deprived	961	32.8	26.4, 39.2	9.2	5.4, 18.7	14.2	12.2, 16.1	0.026			0.022	0.183
	Not deprived	494	44.4	38.2, 50.6	12.5	6.6, 21.0	15.0	13.7, 16.3		0.9	0.8, 1.0		
MPI Living Standards	Deprived	1144	29.8	23.7, 35.9	8.3	5.1, 17.8	13.0	11.1, 14.9	<0.001			0.383	0.036
	Not deprived	413	51.7	44.5, 58.8	15.2	8.9, 22.8	17.3	15.6, 19.0		1.1	0.9, 1.2		
Heard of iodine deficiency	No	827	34.2	27.8, 40.7	9.5	5.6, 19.2	14.0	12.2, 15.9	0.106			0.609	0.584
	Yes	739	41.8	35.5, 48.1	11.5	6.1, 20.5	15.1	13.5, 16.7		1.0	0.9, 1.1		
Heard of iodised salt	No	326	25.0	16.2, 33.8	7.9	5.1, 14.8	12.7	9.6, 15.8	0.013			0.847	0.062
	Yes	1239	41.6	36.1, 47.1	11.5	6.1, 20.8	15.1	13.7, 16.5		1.0	0.9, 1.2		
Salt obtained in sealed pack	No	764	27.8	20.0, 35.5	7.9	4.8, 15.9	11.9	10.1, 13.7	<0.001			0.643	0.024
	Yes	754	43.3	37.2, 49.4	11.7	6.9, 21.6	16.2	14.4, 18.0		1.0	0.9, 1.2		
Salt package had iodine logo or label ^1^	No	225	47.8	37.1, 58.5	14.1	7.6, 22.5	17.5	14.3, 20.8	<0.001			0.468	0.186
	Yes	401	46.9	40.3, 53.4	13.5	7.6, 22.8	17.3	15.4, 19.1		1.0	0.9, 1.3		
	Missing/Don’t know	940	27.6	20.9, 34.3	8.0	4.9, 15.9	11.7 ^a^	10.2, 13.2		0.9	0.7, 1.2		
Salt Brand ^1^	No brand	239	45.3	34.1, 56.4	14.1	7.7, 22.5	17.2	14.2, 20.2	<0.001			0.292	0.713
	Other brand	182	42.9	32.2, 53.6	11.5	6.4, 21.5	16.6	13.5, 19.7		0.9	0.7,1.1		
	Leading market brand	90	56.7	42.1, 71.3	17.4	7.8, 23.9	18.4	15.5, 21.2		1.1	0.8,1.4		
	Missing/Don’t know	1055	31.3	25.3, 37.3	8.9	5.3, 17.6	12.7 ^a^	11.4, 14.0		0.9	0.8,1.1		
Respondent looked for iodised salt purchase	No	1004	31.8	25.3, 38.2	8.6	5.4, 18.1	13.1	11.2, 15.0	<0.001			0.083	0.818
	Yes	435	50.5	44.3, 56.7	15.2	8.4, 22.8	17.9 ^a^	16.0, 19.7		1.2 ^a^	1.0, 1.3		
	Missing/Don’t know	127	23.4	12.0, 34.8	7.5	4.8, 12.5	10.7	8.6, 12.8		1.0	0.8, 1.2		
Grain type	Coarse	1237	30.3	24.1, 36.4	8.4	5.1, 17.6	12.2	10.9, 13.4	<0.001			<0.001	0.011
	Fine	311	51.5	42.0, 61.1	15.6	9.0, 24.3	19.5	16.3, 22.7		1.3	1.2, 1.5		

CI, confidence interval. HH, household. MPI, multi-dimensional poverty index. IQR, inter-quartile range. ^a^ Superscript letter indicates a significant difference *p* < 0.05 to the reference value (first listed level), for variables with more than 2 levels. ^1^ Only asked where salt reported to be obtained in a sealed packet.
